# Impact of Diabetes-Specific Nutritional Formulas versus Oatmeal on Postprandial Glucose, Insulin, GLP-1 and Postprandial Lipidemia

**DOI:** 10.3390/nu8070443

**Published:** 2016-07-22

**Authors:** Adham Mottalib, Barakatun-Nisak Mohd-Yusof, Mohamed Shehabeldin, David M. Pober, Joanna Mitri, Osama Hamdy

**Affiliations:** 1 Joslin Diabetes Center, Boston, MA 02215, USA; Barakatun.Nisak-Mohd-Yusof@joslin.harvard.edu (B.-N.M.-Y.); moshehabeldin@gmail.com (M.S.); david.pober@joslin.harvard.edu (D.M.P.); joanna.mitri@joslin.harvard.edu (J.M.); 2 Department of Nutrition and Dietetics, Faculty of Medicine and Health Sciences, Universiti Putra Malaysia, Selangor 43400, Malaysia

**Keywords:** clinical nutrition, nutritional formula, meal replacement, medical food, diabetes, obesity

## Abstract

Diabetes-specific nutritional formulas (DSNFs) are frequently used as part of medical nutrition therapy for patients with diabetes. This study aims to evaluate postprandial (PP) effects of 2 DSNFs; Glucerna (GL) and Ultra Glucose Control (UGC) versus oatmeal (OM) on glucose, insulin, glucagon-like peptide-1 (GLP-1), free fatty acids (FFA) and triglycerides (TG). After an overnight fast, 22 overweight/obese patients with type 2 diabetes were given 200 kcal of each of the three meals on three separate days in random order. Blood samples were collected at baseline and at 30, 60, 90, 120, 180 and 240 min. Glucose area under the curve (AUC_0–240_) after GL and UGC was lower than OM (*p* < 0.001 for both). Insulin positive AUC_0–120_ after UGC was higher than after OM (*p* = 0.02). GLP-1 AUC_0–120_ and AUC_0–240_ after GL and UGC was higher than after OM (*p* < 0.001 for both). FFA and TG levels were not different between meals. Intake of DSNFs improves PP glucose for 4 h in comparison to oatmeal of similar caloric level. This is achieved by either direct stimulation of insulin secretion or indirectly by stimulating GLP-1 secretion. The difference between their effects is probably related to their unique blends of amino acids, carbohydrates and fat.

## 1. Introduction

Lifestyle changes through diet and increased physical activity are recommended as the first line therapy for patients with type 2 diabetes [1]. In 1994, the American Dietetic Association used the term “Medical Nutrition Therapy” (MNT) to encompass the utilization of proper nutrition for diabetes management [2]. It has been shown that use of diabetes-specific nutritional formulas (DSNFs) as part of MNT was associated with improved postprandial glycemic control [3]. In addition, DSNFs offer structured nutritional alternative that enhances adherence to lifestyle interventions and subsequently lead to meaningful reduction in hemoglobin A1C and body weight [4,5].

Glucagon-like peptide-1 (GLP-1) is an incretin hormone secreted by the intestinal L-cells in response to food consumption [6]. It plays a major role in glucose homeostasis through stimulating pancreatic β-cells secretion of insulin and inhibiting α-cells secretion of glucagon [6]. It also slows gastric emptying and induces satiety [7,8]. Previous studies showed that patients with type 2 diabetes have lower postprandial GLP-1 level in comparison to healthy individuals [9]. Increasing GLP-1 level by incretin-based therapies has become a therapeutic option for patients with type 2 diabetes [10,11,12].

While DSNFs have been shown to improve postprandial glycemic response and increase GLP-1 secretion [13,14], no head-to head comparison was done between commercially available DSNFs versus oatmeal (OM). OM has long been recognized as a healthy breakfast choice for patients with diabetes for having high fiber content [15], which is known to improve glycemic control and enhance insulin response in patients with type 2 diabetes [16,17].

This study was designed to evaluate the effects of two commercially available DSNFs; Glucerna (Abbott Nutrition Inc., Columbus, OH, USA) and Ultra Glucose Control (Metagenics Inc., Aliso Viejo, CA, USA) versus OM (Quaker Oats Co., Chicago, IL, USA) on PP plasma glucose, serum insulin, serum active GLP-1 hormone, serum free fatty acids (FFA) and serum triglycerides (TG) in overweight and obese patients with type 2 diabetes.

## 2. Experimental Section

### 2.1. Study Design and Subjects

**Figure 1:**
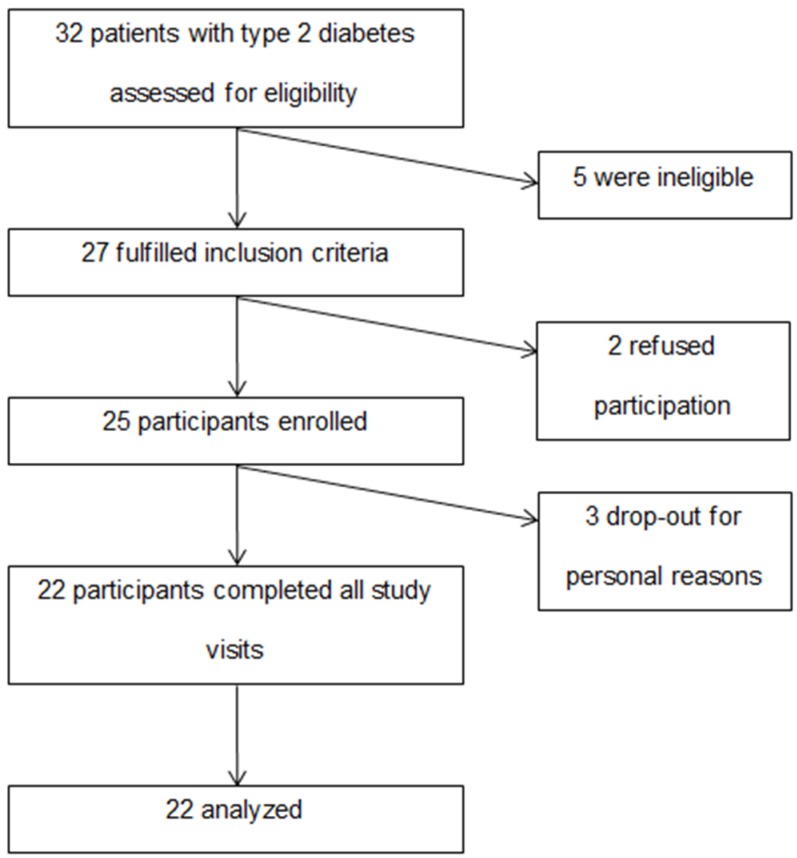
Flow diagram of study enrollment.

This was a cross-over, three-way and open-label clinical study conducted at Joslin Diabetes Center, Boston, MA, USA. The institution’s Committee on Human Studies approved the study protocol and participants signed its informed consent prior to enrollment. Trial Registration: NCT02691481 clinicaltrials.gov. Eligible participants were patients diagnosed with type 2 diabetes for ≥3 months, between age 18 and 75 years, BMI ≥ 25 kg/m^2^, hemoglobin A1C ≥ 6.5% and doses of anti-hyperglycemic or lipid lowering medications have to be stable for ≥3 months. Major study exclusion criteria included use of insulin or GLP-1 analogues for diabetes management, history of bariatric surgery, gastroparesis, malabsorption syndromes and pregnancy or lactation. Thirty-two patients with type 2 diabetes were assessed for eligibility. Five were ineligible due to the use of insulin and/or GLP-1 analogues or having a BMI < 25 kg/m^2^. Twenty-seven met our inclusion criteria from which 2 refused participation prior to signing the informed consent form. Twenty-five participants were enrolled, from which 22 participants completed all study visits (Figure 1). One participant dropped-out due to personal reasons prior to attending any study visit. Two participants dropped-out due to finding visit length and frequent blood drawing inconvenient, after each completing 1 study visit. Data from the three dropped-outs were excluded from our analysis. The mean age ± standard deviation (SD) of the 22 participants was 62.3 ± 6.8 years. Participants’ mean A1C ± SD was 6.8% ± 0.7% and mean diabetes duration ± SD was 9.5 ± 9.8 years. Demographic data of the study participants are shown in Table 1.

### 2.2. Study Procedures

Participants underwent the study procedures on three separate visits. Visits were conducted in the morning after an overnight fast of at least 8 h. The sequence of the three meals was randomly assigned with a washout period of at least two days between visits. Study visits were completed within a three-week window starting from the day of the first visit. This was done to minimize any variability in dietary and exercise patterns. The background diet of the participants was not controlled. However, a 24-h food log was reviewed by a registered dietitian at each visit to confirm the consistency of dietary patterns. Participants were instructed come fasting for at least 8 h and to withhold their anti-hyperglycemic and lipid lowering medications on the morning of the visit. Those treated with dipeptidyl peptidase-4 inhibitors (DPP-4 inhibitors) were asked not to take them for two days prior to each visit. One of three meals (GL, UGC and OM) was served for breakfast at each visit. All meals were equal in caloric content (200 kcal/meal). UGC was prepared by dissolving its powder in 10 fl oz (296 mL) of water; GL was served in the form of 8 fl oz bottles; and OM was prepared by adding 8 fl oz (237 mL) of water to 56 grams of dry oats (Quaker Old Fashioned Oats, Quaker Oats Co., Chicago, IL, USA) then cooking it on a stove for 5–10 min. No milk, sugar or flavoring was added to the oatmeal. Macronutrient composition of each meal is shown in Table 2 and amino acids composition is shown in Table 3.

To ensure safety, participants were asked to check their blood glucose level before coming to each study visit. If it was <70 mg/dL or >300 mg/dL; participants were asked to break their fast, take their diabetes medications then call the study team to reschedule the visit. In addition, blood pressure and capillary blood glucose were measured at the beginning of the visit for safety. If blood glucose was between 70 and 300 mg/dL, a venous line was inserted and a baseline blood sample was drawn. This was followed by consumption of the breakfast meal within 3–5 min. Blood samples were collected at 30, 60, 90, 120, 180 and 240 min from the start of the meal. Blood samples were used to measure plasma glucose; serum insulin, serum active GLP-1, serum free fatty acids and serum triglycerides. After collection of the last sample, participants were given a snack to eat and were instructed to resume their regular diabetes medications.

### 2.3. Statistical Analyses

Values for measured variables are presented as mean ± standard error of mean (SEM) unless otherwise specified. All study quantitative, qualitative and clinical data were collected in a single database for statistical analysis. Data analysis was done using SPSS statistical software (SPSS Institute Inc., Cary, NC, USA). Per-protocol analysis was performed using repeated measures analysis of variance with treatment and time as fixed effects and subject as random effect. Pairwise differences of the treatment means were tested using Tukey-Kramer *p*-value adjustments. If the parametric approach is determined to be inappropriate by the result of Shapiro-Wilk test, then three pairwise treatment differences were analyzed using signed rank test with stepdown Bonferroni *p*-value adjustments. Outcomes were defined as area under the curve between 0 and 120 min and between 0 and 240 min for measured variables over time (AUC_0–120_ and AUC_0–240_) calculated using the trapezoidal formula [18]. Positive AUC was calculated using the same formula but representing the area above the fasting level. The primary endpoint was postprandial blood glucose area under the curve between 0 and 240 min (AUC_0–240_).

### 2.4. Sample-Size Calculation

In order to detect a mean difference of 20% (SD equal to the magnitude of the difference) in the glucose AUC_0–240_ with power of 0.90 and assuming a significance level of 0.025 and a modest correlation (0.60) among repeated measurements within subjects, we estimated that a sample size of 20 participants was required for this study. Anticipating a 25% attrition rate, we recruited 25 participants.

## 3. Results

**Figure 2:**
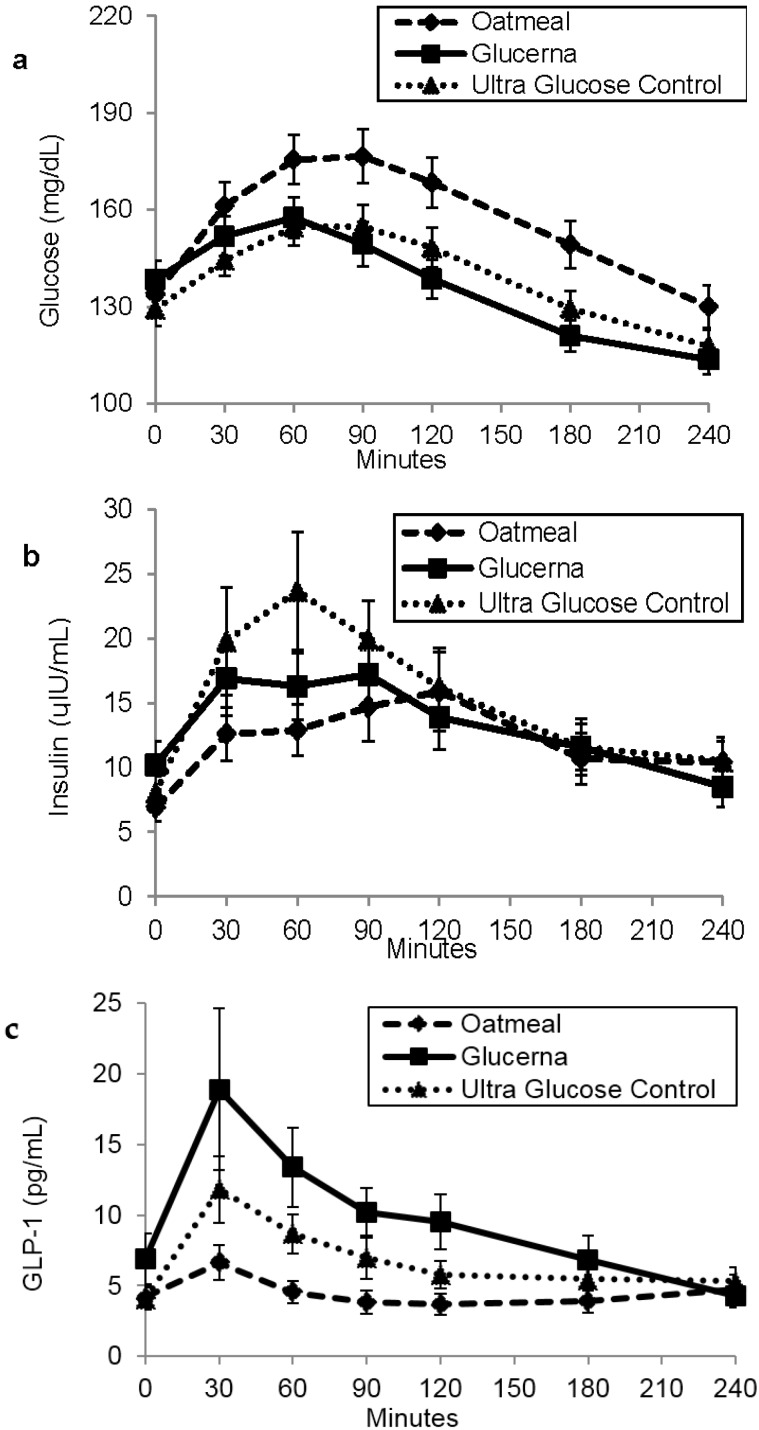
Postprandial glucose (**a**); insulin (**b**); GLP-1 (**c**) levels in response to the breakfast meals. Values are mean ± SEM.

Mean fasting plasma glucose levels at baseline for OM, GL and UGC were comparable (133.8 ± 6.6, 138.4 ± 5.9 and 129.2 ± 5.1 mg/dL respectively) (Figure 2a). Glucose AUC_0–120_ and AUC_0–240_ for GL and UGC were significantly lower than that of OM (*p* < 0.001 for all) (Table 4). There was no significant difference in glucose AUC_0–120_ or AUC_0–240_ when comparing GL to UGC (*p* = 0.98 and *p* = 0.18 respectively). Glucose positive AUC_0–120_ and positive AUC_0–240_ for GL were significantly lower than OM and UGC (*p* < 0.001 for all). Glucose positive AUC_0–120_ and positive AUC_0–240_ for UGC was significantly lower than OM (*p* < 0.001 for both).

**Figure 3:**
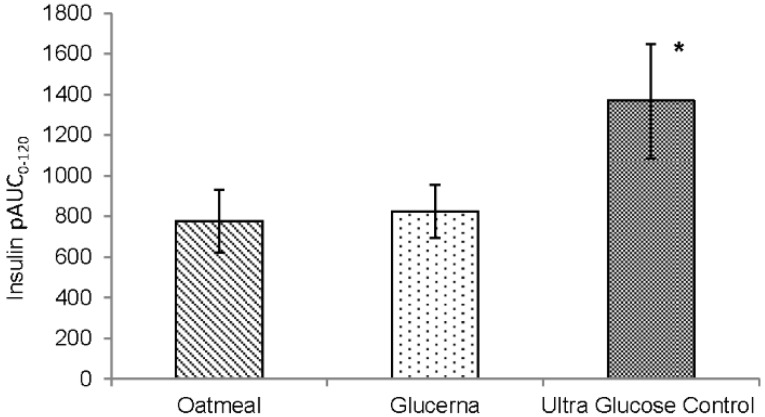
Postprandial insulin positive area under the curve 0–120 min (pAUC_0–120_) in response to the breakfast meals. Values are mean ± SEM. * *p* < 0.05 compared to oatmeal.

Mean baseline fasting serum insulin levels before OM, GL and UGC were also comparable (6.9 ± 1.1, 10.2 ± 1.2 and 8.0 ± 1.3 µIU/mL respectively) (Figure 2b). Insulin AUC_0–120_ and AUC_0–240_ showed no significant difference between any of the breakfast meals. However, insulin positive AUC_0–120_ for UGC was significantly higher than that of OM (*p* = 0.02) while there was no significant difference between GL and OM, or between GL and UGC (Figure 3). Insulin positive AUC_0–240_ showed no significant difference between any of the breakfast meals (Table 4).

Mean baseline fasting values for serum active GLP-1 before OM, GL and UGC were comparable (4.18 ± 0.81, 6.91 ± 1.77 and 4.08 ± 0.78 pg/mL, respectively) (Figure 2c). Serum active GLP-1 AUC_0–120_ and AUC_0–240_ after GL was significantly higher than after OM (*p* < 0.001 for both) and after UGC (*p* = 0.009 and *p* = 0.024, respectively). GLP-1 AUC_0–120_ and AUC_0–240_ for UGC was also higher than after OM (*p* < 0.001 for both).

**Figure 4:**
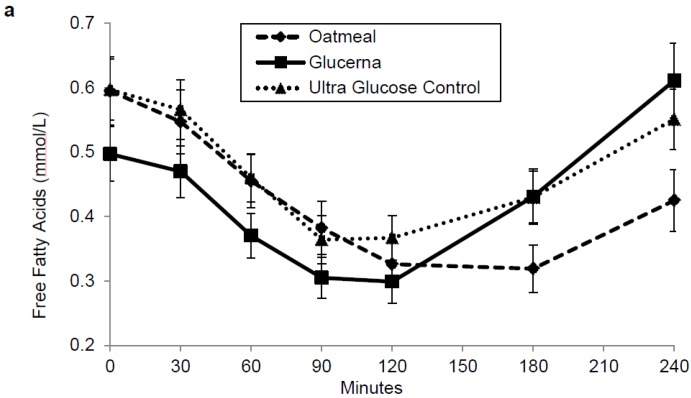

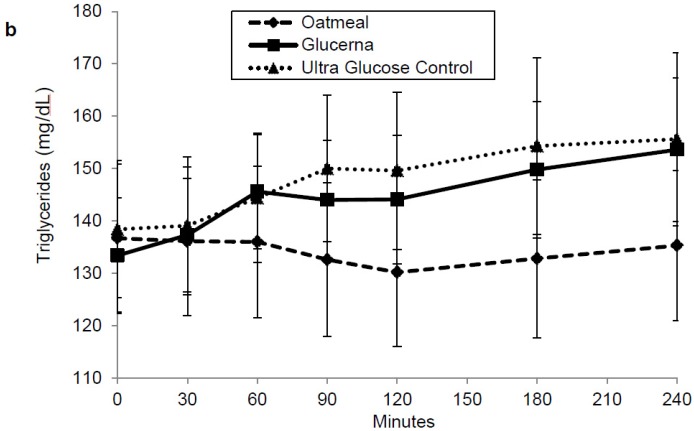
Postprandial free fatty acids (**a**) and triglycerides (**b**) in response to the breakfast meals. Values are mean ± SEM.

Mean baseline fasting serum free fatty acid before OM, GL and UGC at baseline were comparable (0.6 ± 0.05, 0.5 ± 0.04 and 0.6 ± 0.05 mg/dL, respectively). Free fatty acids AUC_0–120_ and AUC_0–240_ showed no significant difference between any of the breakfast meals (Figure 4a). Mean baseline fasting serum triglycerides before OM, GL and UGC at baseline were comparable (136.7 ± 14.1, 133.4 ± 11.0 and 138.4 ± 13.1 mg/dL, respectively). Triglycerides AUC_0–120_ and AUC_0–240_ showed no significant difference between any of the breakfast meals (Figure 4b).

## 4. Discussion

This study shows that postprandial plasma glucose is significantly lower after either of the two DSNFs in comparison to OM. This observation is in line with previous studies which used similar formulas [13,14,19]. The low glycemic index of most DSNFs partially contributes to this effect [14,20]. Lowering postprandial glucose was shown to reduce the need for anti-hyperglycemic medications [1] and was associated with reduced risk of diabetes complications [21,22].

OM is frequently recommended for patients with diabetes for being rich in polysaccharide β-glucan, which was found to reduce postprandial plasma glucose levels. This makes it an ideal breakfast for patients with diabetes [16,17,23]. However, this study showed that plasma glucose returns back to baseline within a shorter period following DSNFs in comparison to OM. This was achieved within 120 min after GL, 180 min after UGC and within 240 min after OM breakfast (Figure 2a). This effect is particularly important in diabetes management since prolonged excursion of postprandial plasma glucose was shown to be associated with overproduction of oxygen free radicals, vasoconstriction and increased circulating levels of pro-inflammatory cytokines that may contribute to cardiovascular complications [24,25]. The amount of β-glucan in the commonly served OM breakfast, similar to the amount tested in this study, is relatively low (1.3 g per serving). A previous study showed that 6 grams of β-glucan is required to induce a 10% reduction in postprandial glycemic responses [17].

Protein intake was shown to increase postprandial insulin levels especially during its early phase of secretion [26,27]. First phase insulin secretion, which is frequently reduced or lost in patients with long standing type 2 diabetes, is critical in reducing postprandial glucose exertion [28]. Both DSNFs contain higher amount of protein with some differences in their amino acids ratio. This study showed that insulin secretion is significantly higher for 120 min after UGC in comparison to OM. This difference was not seen between GL and OM. This observation may be explained by the difference in amount and type of branched chain amino acids between the two formulas (Table 3). Leucine and phenylalanine, which are higher in UGC, are among amino acids with the highest insulinotropic effect [27,29] (Table 3). This finding is particularly important since reduced early phase insulin secretion leads to prolonged PP hyperglycemia in patients with type 2 diabetes [28,30]. It was presumed that β-cells response to ingested nutrients is either lost or significantly weakened in patients with long duration of type 2 diabetes, as in the case of this study’s participants. However, this study showed that pancreatic β-cells retained their response to specific amino acids.

GLP-1 secretion in response to a meal is directly related to its macronutrient composition, notably its content of carbohydrate and fat [6]. This study showed that active GLP-1 AUC_0–120_ is significantly higher after GL in comparison to OM and UGC and after UGC in comparison to OM. It was previously shown that high dietary monounsaturated fat (MUFA) increases postprandial GLP-1 levels without a significant effect on insulin levels [31,32]. MUFA content of GL is higher than that of UGC and OM (Table 2). This difference might explain the difference in GLP-1 response. It also confirms previous observations that the amount and the type of fat and carbohydrates play important role in promoting GLP-1 secretion [13,14].

Postprandial FFA levels are usually reduced by lowering glycemic load of a meal (amount of carbohydrate × glycemic index) [30]. Although glycemic load of both DSNFs is much lower than that of OM, there were no significant differences in postprandial FFA levels between the three meals. Similarly, there was no difference in postprandial serum triglycerides response to the three meals. These observations are most probably related to the higher fat content of DSNFs, which possibly muted the benefit of their low glycemic load on postprandial lipidemia.

We should be cautious in interpreting the results of this study as it has several limitations. As shown, it was primarily conducted to evaluate the mechanisms of action of DSNFs. Its cross-sectional design limits the value of its positive results from suggesting any long-term benefits of DSNFs in improving A1C or body weight. A longitudinal randomized controlled clinical study is needed to prove that these beneficial postprandial effects may be translated into a meaningful long-term glycemic control and/or weight reduction. Due to major difference in texture between OM and DSNFs, we were not able to blind participants to the three meals. However, we think non-blinded approach has a minimal effect on this study’s results. While oatmeal used in this study was prepared with water only, many may prepare and/or consume it with different foods/beverages. How these ingredients may affect postprandial metabolic parameters warrants further investigation. This study did not control the background diets of its participants. While the implication of this on the primary endpoint is unknown, we sought to minimize variability by having participants fast for at least 8 h prior to each visit in addition to having each participant complete all study visits within a three-week window. We acknowledge that most study participants were on anti-hyperglycemic medications of which seven were on DPP-4 inhibitors. Since this study had a cross-over design, participants served as their own control; therefore, it is unlikely that medications have unequally affected the study outcomes. Lastly, while caloric content was equal between the three tested meals; carbohydrate content was different (Table 2). Both DSNFs have similar carbohydrate content but is much lower than that of OM. Another study may be needed to evaluate the differences between DSNFs and OM when glycemic load is equal. However, this study may support the concept that macronutrient composition is much important than the caloric value of a meal and that insulin and GLP-1 secretions can be induced at lower glycemic load.

## 5. Conclusions

This study shows that DSNFs are superior to the common American breakfast of OM in reducing postprandial glucose response in overweight and obese patients with type 2 diabetes. This effect is achieved either through direct stimulation of insulin secretion from pancreatic β-cells or indirectly through stimulation of GLP-1 production. The differences between their effects on insulin and GLP-1 hormones are probably related to their unique blends of amino acids, carbohydrates and fat content, which warrants further investigation.

## Figures and Tables

**Table 1:** Characteristics of study participants (*n* = 22).

Gender	
Male	12 (54.6%)
Female	10 (45.5%)
Age (years)	62.3 ± 6.8
Height (cm)	171.1 ± 8.9
Weight (kg)	97.4 ± 21.3
BMI (kg/m^2^)	33.2 ± 5.9
Diabetes duration (years)	9.5 ± 9.8
HbA1c (%)	6.8 ± 0.7
Participants using diabetes medications (*n*)	19
Participants using DPP-4 inhibitors	6
Participants using lipid lowering medications	15

Gender *n* (%). Values are mean ± standard deviation. Dipeptidyl peptidase-4 inhibitors (DPP-4 inhibitors).

**Table 2:** Nutrition information of the three breakfast meals.

	Oatmeal (OM)		Glucerna (GL)		Ultra Glucose Control (UGC)	
	Amount	% DV	Amount	% DV	Amount	% DV
Serving Size	53.3 (g)	NA	8 (fl oz)	NA	56 (g)	NA
Energy (kcal)	200	10	200	10	200	10
Total Fat (g)	4	6.7	7	11	7	11
% Energy	18	-	32	-	32	-
Saturated Fat (g)	0	0	0.5	3	1	5
Monounsaturated Fat (g)	1.3	-	5.2	-	4.5	-
Total Carbohydrates (g)	36	12	26	9	27	9
% Energy	72	-	52	-	54	-
Dietary Fiber (g)	5.3	20	3	12	3	12
Protein (g)	6.7	NA	10	20	15	30
% Energy	13	-	20	-	30	-

% DV: percentage daily values were calculated based on a 2000 kcal diet. Oatmeal (OM, Quaker Oats Co., Chicago, IL, USA); Glucerna (GL, Abbott Nutrition Inc., Columbus, OH, USA); Ultra Glucose Control (UGC, Metagenics Inc., Aliso Viejo, CA, USA).

**Table 3:** Amino acids composition of the three breakfast meals.

Amino Acid	Oatmeal ^a^	Glucerna ^b^	Ultra Glucose Control ^b^
Histidine	0.164	0.327	0.329
Isoleucine	0.295	0.603	1.119
Leucine	0.567	1.169	1.850
Lysine	0.290	0.946	0.872
Methionine	0.131	0.312	0.175
Phenylalanine	0.391	0.610	0.731
Threonine	0.259	0.655	0.518
Tryptophan	-	0.154	0.145
Valine	0.398	0.733	1.183
Alanine	0.354	0.442	0.613
Arginine	0.491	0.473	1.167
Aspartic acid	0.602	1.027	1.492
Cystine	0.208	0.076	0.160
Glutamic acid	1.635	2.650	2.261
Glycine	0.367	0.274	0.560
Proline	0.405	1.126	0.604
Serine	0.367	0.710	0.698
Tyrosine	0.257	0.544	0.540

Values are in grams per serving. ^a^ Source: Amino-Acid Content of Foods and Biological Data on Proteins, Food and Agriculture Organization of the United Nations reference profile; ^b^ Source: direct communication with the manufacturers.

**Table 4:** Area under the curve 0–240 min for the different variables in response to the meals.

	Oatmeal (OM)	Glucerna (GL)	Ultra Glucose Control (UGC)
Glucose (mg·min/dL)	37,828.6 ± 1678.7	32,747.0 ± 1287.7 *	33,538.6 ± 1266.5 *
Insulin (IU·min/mL)	2995.1 ± 442.4	3247.3 ± 538.9	3799.6 ± 620.9
Active GLP-1 (pg·min/mL)	1055.5 ± 187.3	2347.7 ± 464.2 *	1631.5 ± 246.8 *
Free fatty acids (mmol·min/L)	97.023 ± 8.7	99.2 ± 8.5	108.4 ± 9.0
Triglyceride (mg·min/dL)	32,077.5 ± 3517.6	34,887.3 ± 2857.0	35,739.5 ± 3438.4

Values are mean ± SEM. * *p* < 0.001 compared to oatmeal.

## Abbreviations

The following abbreviations are used in this manuscript:

AUC	area under the curve
DSNFs	diabetes-specific nutritional formulas
FFA	free fatty acids
GLP-1	glucagon-like peptide-1
GL	Glucerna
MNT	medical nutrition therapy
MUFA	monounsaturated fatty acids
OM	oatmeal
PP	postprandial
SEM	standard error of mean
TG	triglycerides
UGC	Ultra Glucose Control
